# Neuraminidase Inhibitor Susceptibility Testing in Human Influenza Viruses: A Laboratory Surveillance Perspective

**DOI:** 10.3390/v2102269

**Published:** 2010-10-13

**Authors:** Margaret Okomo-Adhiambo, Katrina Sleeman, Kristina Ballenger, Ha T. Nguyen, Vasiliy P. Mishin, Tiffany G. Sheu, James Smagala, Yan Li, Alexander I. Klimov, Larisa V. Gubareva

**Affiliations:** 1 Virus Surveillance and Diagnosis Branch, Influenza Division, National Center for Immunization and Respiratory Diseases, Centers for Disease Control and Prevention, 1600 Clifton Rd NE, Mailstop G16, Atlanta, GA 30333, USA; E-Mails: gfv3@cdc.gov (M.O.-A.); hhk6@cdc.gov (K.S.); isx0@cdc.gov (K.B.); hsn7@cdc.gov (H.T.N.); fwc7@cdc.gov (V.P.M.); gbq3@cdc.gov (T.G.S.); gpa1@cdc.gov (J.S.); axk0@cdc.gov (A.I.K.); 2 Atlanta Research and Education Foundation, 1670 Clairmont Rd, 151F, Decatur, GA 30033, USA; 3 Battelle, Century Plaza 1, 2987 Clairmont Rd, Suite 450, Atlanta, GA 30329, USA; 4 Oak Ridge Institute for Science and Education, MC-100-22, P.O. Box 117, Oak Ridge, TN 37831, USA; 5 Influenza and Respiratory Viruses Section, National Microbiology Laboratory, Public Health Agency of Canada, 1015 Arlington St., Suite H4050, Winnipeg, MB, R3E 3R2, Canada; E-Mail: yan.li@phac-aspc.gc.ca

**Keywords:** Oseltamivir, zanamivir, peramivir, seasonal influenza A and B, pandemic H1N1

## Abstract

Neuraminidase inhibitors (NAIs) are vital in managing seasonal and pandemic influenza infections. NAI susceptibilities of virus isolates (n = 5540) collected during the 2008–2009 influenza season were assessed in the chemiluminescent neuraminidase inhibition (NI) assay. Box-and-whisker plot analyses of log-transformed IC_50_s were performed for each virus type/subtype and NAI to identify outliers which were characterized based on a statistical cutoff of IC_50_ >3 interquartile ranges (IQR) from the 75^th^ percentile. Among 1533 seasonal H1N1 viruses tested, 1431 (93.3%) were outliers for oseltamivir; they all harbored the H275Y mutation in the neuraminidase (NA) and were reported as oseltamivir-resistant. Only 15 (0.7%) of pandemic 2009 H1N1 viruses tested (n = 2259) were resistant to oseltamivir. All influenza A(H3N2) (n = 834) and B (n = 914) viruses were sensitive to oseltamivir, except for one A(H3N2) and one B virus, with D151V and D197E (D198E in N2 numbering) mutations in the NA, respectively. All viruses tested were sensitive to zanamivir, except for six seasonal A(H1N1) and several A(H3N2) outliers (n = 22) which exhibited cell culture induced mutations at residue D151 of the NA. A subset of viruses (n = 1058) tested for peramivir were sensitive to the drug, with exception of H275Y variants that exhibited reduced susceptibility to this NAI. This study summarizes baseline susceptibility patterns of seasonal and pandemic influenza viruses, and seeks to contribute towards criteria for defining NAI resistance.

## 1. Introduction

Antiviral drugs play an essential role in the management of infections caused by seasonal and pandemic influenza viruses. Adamantanes (M2 ion channel blockers) and neuraminidase inhibitors (NAIs) are two classes of drugs licensed for prevention or treatment of influenza A virus infections [1], however, the effectiveness of adamantanes is compromised by resistance among seasonal influenza A(H3N2) and A(H1N1) viruses circulating in certain geographic areas [2,3].

Two FDA-approved NAIs, oseltamivir and zanamivir, are presently the only antiviral drugs that are effective for the treatment and chemoprophylaxis of seasonal as well as 2009 H1N1 pandemic influenza infections. An investigational NAI, peramivir, developed as an intravenous (IV) formulation, was recently prescribed in the United States under an emergency use authorization (EUA) for treatment of 2009 pandemic influenza H1N1 infection in certain patients [4] and is now licensed in Japan [5]. Another neuraminidase inhibitor, R-125489, is being developed by Daiichi Sankyo and Biota as an inhaled prodrug, laninamivir (CS-8958). NAIs competitively bind to the highly conserved NA active site which comprises of catalytic and framework residues [6]. Mutations at this site arise from single amino acid changes [7] which confer resistance to NAIs in a drug-and virus type/NA subtype-specific manner.

Prior to 2007, resistance to NAIs among circulating influenza viruses was low (<1% worldwide) [8–10]. However, the 2007–2008 influenza season was marked by an emergence of oseltamivir-resistant seasonal influenza A(H1N1) viruses with the H275Y (H274Y in N2 numbering) mutation in the NA, which was first reported to the World Health Organization (WHO) by Norway in late January 2008 [11]. In the United States, 11% of all 2007–2008 seasonal influenza A(H1N1) viruses were resistant to oseltamivir [10,12], while the prevalence of oseltamivir resistance was especially high in some European countries [13,14] and in Africa [15], emphasizing the critical need for NAI susceptibility surveillance of influenza viruses circulating globally.

Early 2008–2009 influenza season surveillance data suggested that oseltamivir resistance among seasonal influenza A(H1N1) viruses would most likely be higher than in the previous 2007–2008 season [16]. In the present study, oseltamivir resistance was observed in 93% of seasonal influenza A(H1N1) virus isolates circulating globally during the 2008–2009 season, with many countries reporting up to 100% oseltamivir resistance [17,18].

Assessment of NAI susceptibility is primarily performed in functional neuraminidase inhibition (NI) assays that allow detection of drug-resistant viruses with established mutations (e.g. H275Y in N1 subtypes) and/or novel mutations. The most commonly used NI assays, the chemiluminescent [8,10,19,20] and fluorescent [21,22], both utilize small synthetic substrates [23,24] and generate IC_50_ values (drug concentration inhibiting NA activity by 50%) that are used to characterize NAI susceptibility. Both assays require the use of grown viruses, however, commonly used cell cultures such as the Madin-Darby canine kidney (MDCK) cells have been shown in some instances, to provide a growth advantage to particular virus variants, including those with mutations in the NA and thus may alter virus susceptibility after propagation [25,26].

Criteria defining NAI resistance were set by the Global Neuraminidase Inhibitor Susceptibility Network (NISN) as either IC_50_ >3SD from the mean (or median) or IC_50_ >10-fold mean (or median) for the influenza type/subtype and drug [27]. Resistant isolates with known and characterized drug resistance conferring mutations tend to have IC_50_ values 100–10,000 times higher than the normal range for that type/subtype and are clearly recognizable based on their NAI profiles. IC_50_ values are affected and vary by NI assay type, influenza virus type/subtype, and drug tested [20,21,28], requiring specific criteria for NAI resistance to be drawn for each factor. Nevertheless, the resulting IC_50_ values provide valuable information for detection of resistant viruses, as well as for comparison of inhibitory effects of different NAIs. The clinical relevance of the resistance detected using the NA inhibition assay alone has not been sufficiently evaluated, therefore, it is essential to monitor genetic changes in the NA and their possible effect on virus susceptibility to existing NAIs.

The definitive characterization of NAI resistance combines elevated IC_50_ values with the presence of established molecular markers of resistance in the NA as determined by pyrosequencing [29,30] or conventional Sanger sequencing [10,21]. However, the latter criterion is hindered by insufficient information on markers of NAI resistance, which are not yet fully characterized. Molecular-based methods such as real time PCR [31,32], sequencing [10,21] or pyrosequencing [29,30] may offer rapid techniques for the detection of known resistance mutations, but will not identify novel mutations that may confer resistance, or identify subtle differences in the susceptibility of viruses to NAIs.

For the purpose of this study, analyzed viruses included those collected during the time period (start day – October 1, and end day – September 30) commonly referred to as an influenza season. The study presents NAI surveillance data of seasonal influenza A (H1N1 and H3N2) and B viruses collected globally from October 01, 2008 to September 30, 2009, as well as the 2009 pandemic H1N1 viruses collected from April 2009 through September 30, 2009. This study defines baseline susceptibility patterns of seasonal and pandemic influenza viruses, and seeks to contribute further criteria for evaluating NAI resistance.

## 2. Results and Discussion

### 2.1. Susceptibility of seasonal influenza virus isolates to neuraminidase inhibitors

The 2008–2009 influenza season was unlike previous seasons, due to the high prevalence of oseltamivir resistance reported among seasonal influenza A(H1N1) viruses [17,18] and the emergence of the 2009 pandemic H1N1 virus in April 2009 [33]. Seasonal and pandemic influenza virus isolates (n = 5540) collected from different geographic regions between October 01, 2008 and September 30, 2009 were passaged (once or twice) in MDCK cells and then routinely tested for oseltamivir and zanamivir sensitivity in the chemiluminescent NI assay. In addition, a subset of viruses (n = 1058) was tested for susceptibility to an investigational NAI, peramivir. Seasonal and pandemic reference viruses representative of each virus type/subtype were used as controls for the NI assay (Table 1).

Of note, since the vast majority of 2008–2009 seasonal influenza A(H1N1) viruses exhibited elevated IC_50_s for oseltamivir when tested in the NI assay, all viruses of this subtype were tested in parallel in the NI assay and by pyrosequencing analysis targeted at residue H275 of the NA, to identify oseltamivir-resistant H275Y variants.

Raw NI assay data were initially analyzed to determine IC_50_ values using curve fitting software, JASPR, which was recently developed at the CDC. The IC_50_ values generated in JASPR (Table 2) were highly correlated with those calculated by Robosage software (in-house, GlaxoSmithKline), which was previously used in NAI susceptibility studies [10,26]. Both programs can be used for curve fitting and IC_50_ calculation, however, JASPR provides a faster and higher throughput method of IC_50_ determination, and has a more user-friendly format.

**Figure 1:**
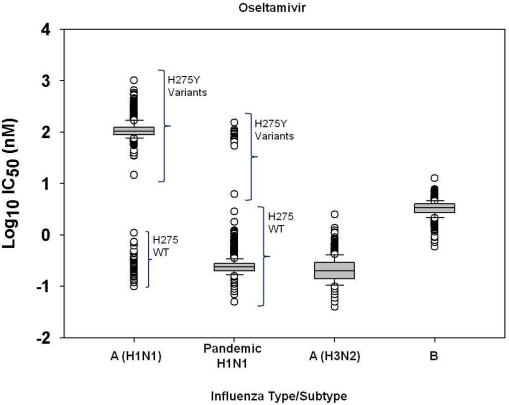
Box-and-whisker plots showing distributions of log-transformed oseltamivir IC_50_ values for seasonal influenza A(H1N1) (n = 1533), 2009 pandemic H1N1 (n = 2259), seasonal influenza A(H3N2) (n = 834), and seasonal influenza B (n = 914) virus isolates. The boxes represent the 25^th^ to 75^th^ percentiles, and horizontal lines within the box represent median values. The whiskers represent the lowest and highest value in the 25^th^ percentile minus 1.5IQR and 75^th^ percentile plus 1.5IQR regions, respectively.

**Figure 2:**
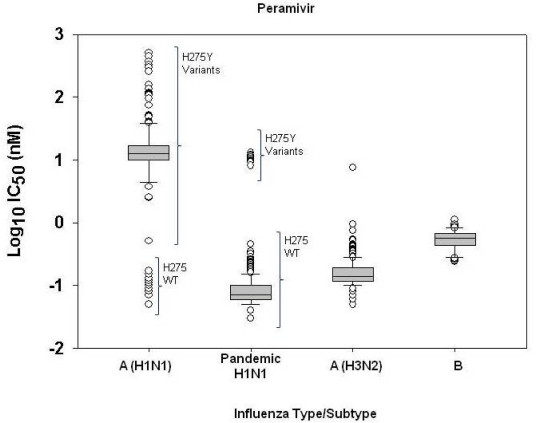
Box-and-whisker plots showing distribution of log-transformed peramivir IC_50_ values for seasonal influenza A(H1N1) (n = 235), 2009 pandemic H1N1 (n = 550), seasonal influenza A(H3N2) (n = 220), and seasonal influenza B (n = 52). The boxes represent the 25^th^ to 75^th^ percentiles, and horizontal lines within the box represent median values. The whiskers represent the lowest and highest value in the 25^th^ percentile minus 1.5IQR and 75^th^ percentile plus 1.5IQR regions, respectively.

**Figure 3:**
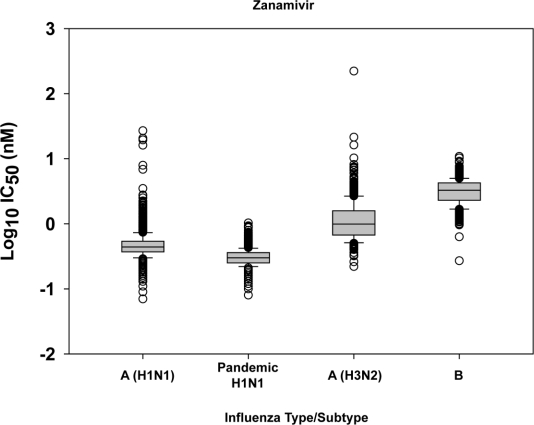
Box-and-whisker plots showing distributions of log-transformed zanamivir IC_50_ values for seasonal influenza A(H1N1) (n = 1533), 2009 pandemic H1N1 (n = 2259), seasonal influenza A(H3N2) (n = 834), and seasonal influenza B (n = 914) virus isolates. The boxes represent the 25^th^ to 75^th^ percentiles, and horizontal lines within the box represent median values. The whiskers represent the lowest and highest value in the 25^th^ percentile minus 1.5IQR and 75^th^ percentile plus 1.5IQR regions, respectively.

**Figure 4:**
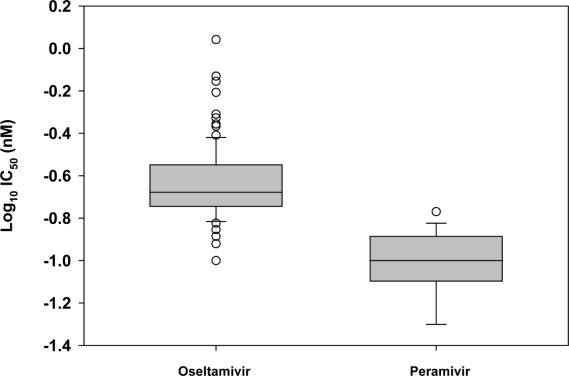
Box-and-whisker plots showing distributions of log-transformed IC_50_ values for oseltamivir carboxylate and peramivir among oseltamivir-sensitive (wildtype H275) seasonal influenza A(H1N1) virus isolates (n = 102). The boxes represent the 25^th^ to 75^th^ percentiles, and horizontal lines within the box represent median values. The whiskers represent the lowest and highest value in the 25^th^ percentile minus 1.5IQR and 75^th^ percentile plus 1.5IQR regions, respectively.

To identify viruses with elevated IC_50_ values, box-and-whisker plot analyses of log-transformed IC_50_ values [8] were performed for each virus type/subtype and respective drug (Figures 1–4). Log-transformation of IC_50_ values was necessary as they are not normally distributed parameters [8]. Previous surveillance studies [8,34] used box-and-whisker plot analyses to identify two kinds of outliers, mild (between 1.5 and 3.0 times the interquartile range (IQR) from the 25^th^ and 75^th^ percentile) and extreme (3.0 times the IQR from the 25^th^ and 75^th^ percentiles and at least >10-fold mean IC_50_). In a similar surveillance study [35], box-and-whisker plot analysis identified outliers as isolates with IC_50_ 1.5 times the IQR from the 25th and 75^th^ percentiles, while in another study [21] isolates with IC_50_ values outside the 95% confidence limits (mean IC_50_ ± 2 SD) were characterized as outliers. One surveillance study [10] used the criterion of “mean IC_50_ value + 3 SD” to identify outliers; extreme outliers were isolates with IC_50_s outside this criterion and >10-fold the mean IC_50_ for each respective type/subtype and drug, and mild outliers were those with IC_50_s outside the cutoff criterion but <10-fold of the mean IC_50_ value. The same study [10] also performed box plot analyses of all isolates by type/subtype and drug, excluding extreme outliers, to determine IQR and to establish a statistical cutoff for isolates with IC_50_s > 3.0 times the IQR to the right of the third quartile (*X*_0.75_) (IC_50_ > *X*_0.75_ + 3 IQR).

In the present study, box-and-whisker plot analyses identified extreme outliers as virus isolates with IC_50_ > *X*_0.75_ + 3IQR and ≥10-fold the mean IC_50_ of the drug for the virus type/subtype. Mild outliers were isolates with IC_50_ > *X*_0.75_ + 3IQR, but >2-fold and <10-fold that of the mean IC_50_ of the drug for the virus type/subtype. These criteria were elected for mild outliers, as using previous criterion of IC_50_ between 1.5 and 3IQR from the 75^th^ percentile [8,34,36] resulted in the characterization of too many isolates without genetic changes in the NA as outliers. Outliers with IC_50_s below the 25^th^ percentile were not characterized, but were considered NAI-susceptible.

In this study, the box-and-whisker plot analysis to determine outliers for oseltamivir among seasonal influenza A(H1N1) virus isolates tested (n = 1533) revealed two diverse clusters of oseltamivir IC_50_s (Figure 1). All viruses with IC_50_s in the upper cluster (n = 1431) harbored the H275Y oseltamivir-resistance conferring mutation in their NA, based on pyrosequencing analysis, and were reported as oseltamivir-resistant, while those with IC_50_s in the lower cluster located 3IQR below the 25^th^ percentile (n = 102) exhibited wildtype sequence at residue H275 of the NA.

The distribution of peramivir IC_50_ values (Figure 2) among seasonal influenza A(H1N1) viruses (n = 235) was similar to that of oseltamivir IC_50_s for this subtype (Figure 1), with a distinct separation of H275Y variants (n = 216) and H275 wildtype viruses (n = 19). However, the distribution of zanamivir IC_50_ values (Figure 3) among seasonal influenza A(H1N1) virus isolates tested for the drug (n = 1533) did not distinguish H275Y variants from viruses lacking the mutation.

As a result of the distinct clustering of IC_50_s observed in box-and-whisker plots for oseltamivir and peramivir among seasonal influenza A(H1N1) viruses, it was necessary to perform additional similar analyses on oseltamivir-susceptible (H275 wildtype) viruses alone, for both drugs (Figure 4), in order to set a statistical cutoff and baseline for oseltamivir and peramivir susceptibility for this subtype, and to identify mild outliers lacking H275Y which may harbor novel mutations in the NA. In addition, the descriptive statistics of IC_50_ values for each drug were computed separately for seasonal influenza A(H1N1) H275Y variants and H275 wildtype viruses, given their distinctive drug resistance genotypes and phenotypes (Table 3).

Following box-and-whisker plot analyses for H275 wildtype viruses (Figure 4), statistical cutoffs of 0.58 nM and 0.28 nM for oseltamivir and peramivir, respectively, were determined based on the criterion IC_50_>X_0.75_ + 3IQR (Table 3). All seasonal influenza A(H1N1) H275Y variants were outliers for oseltamivir and peramivir based on this criterion, in addition to having IC_50_≥10-fold mean IC_50_ for the respective drugs. A few outliers for oseltamivir (n = 4) were identified among H275 wildtype seasonal influenza A(H1N1) viruses (Figure 4) including A/Shannxi-Beilin/1264/2008 (H1N1) which showed a mix at residue D151 of the NA (D151D/N). A similar mix (D151D/N) was also observed in the isolate A/Shanghai-Nanhui/1156/08 (H1N1), the only peramivir outlier among the H275 wildtype viruses. These outliers exhibited IC_50_s that were only 2- to 4-fold more than the mean IC_50_ for the respective drugs (Table 3).

The mean IC_50_ for oseltamivir among H275Y variants (123.32 nM) was ∼500-fold greater than that of the H275 wildtype viruses (0.23 nM; Table 3) and the oseltamivir-sensitive control virus, A/Washington/10/2008 (0.23 nM; Table 1), and was 135-fold more than the mean IC_50_ for oseltamivir (0.91 nM) previously published for seasonal influenza A(H1N1) viruses collected between 2004–2007 and also tested in the chemiluminescent NI assay [10]. The mean IC_50_ for peramivir among the H275Y variants (30.56 nM) was ∼300-fold more than that of viruses lacking this mutation (0.10 nM), as well as that of the oseltamivir-sensitive control virus, A/Washington/10/2008 (0.09 nM). A wide variation in IC_50_ values was observed among the H275Y variants (14.71–1023.68 nM), but not the H275 wildtype viruses (0.1–0.49 nM). The isolate, A/England/412/2008 (H1N1), with an IC_50_ of 14.71 nM for oseltamivir exhibited the presence of mixed wildtype and mutant sequences at residue H275 (H275H/Y) following full NA sequence analysis. This isolate also exhibited a lower IC_50_ (0.51 nM) for peramivir compared to other H275Y variants and was excluded from the descriptive statistical analyses of the respective drug IC_50_s among seasonal influenza A(H1N1) H275Y variants. The highest IC_50_ for oseltamivir (1023.68 nM) was observed in the A/Thailand/1035/2008 (H1N1) which exhibited dominant H275Y mutation in addition to a mix of wildtype and mutant sequences at residue D151 (D151D/G). This isolate was also an outlier for zanamivir (20.61 nM) and peramivir (295.21 nM). The IC_50_ values for oseltamivir among seasonal influenza A(H1N1) viruses comprising populations of 100% H275Y variants without mutations at D151 in the NA ranged from 34.69 nM to 657.88 nM.

All seasonal influenza A(H1N1) viruses tested for zanamivir (n = 1533) were sensitive to the drug, with the exception of some outliers (n = 6) among the H275Y variants whose IC_50_s were above the statistical cutoff of 1.05 nM and ≥10-fold mean IC_50_ (0.51 nM) for this group of viruses (Table 3). The outliers included A/Thailand/1035/2008 (H1N1) mentioned above, and A/Hawaii/20/2008 (H1N1), both with H275Y and D151D/G mutations in their NA. The presence of concurrent mutations at NA residues H275 and D151 in seasonal influenza A(H1N1) virus isolates substantially enhances resistance to oseltamivir and peramivir and/or zanamivir, however, the changes at D151 are typically cell-derived and not present in clinical specimens [26].

All seasonal influenza A(H3N2) viruses tested for oseltamivir (n = 834) were sensitive to the drug (Figure 3 and Table 3), with exception of one outlier, A/Ontario/RV0442/2009, with an IC_50_ of 2.50 nM, which was beyond the IC_50_ cutoff (0.74 nM) and ≥10-fold mean IC_50_ for the drug (0.24 nM). A/Ontario/RV0442/2009, which was also the only outlier for peramivir (7.48 nM) among A(H3N2) viruses tested for peramivir (n = 220), was an outlier for zanamivir as well (221.06 nM). Full NA sequence analysis of A/Ontario/RV0442/2009 revealed the presence of a D151V mutation in addition to V50M. The D151V mutation was previously detected in the NA of A/Montana/8/2007 [10] where it resulted in a 150-fold decrease in zanamivir susceptibility compared to a zanamivir-sensitive virus.

The majority of seasonal influenza A(H3N2) viruses were sensitive to zanamivir, with exception of some outliers (n = 3) with IC_50_ beyond the statistical cutoff (4.31 nM) and ≥10-fold mean IC_50_ for the drug (1.23 nM), including the above-mentioned A/Ontario/RV0442/2009, as well as A/Maryland/02/2009 (16.21 nM) and A/Vladivostok/53/2009 (21.31 nM) whose full NA sequences revealed the presence of D151G and mixed D151D/G mutations, respectively. In addition, a few mild zanamivir outliers (n = 19) with IC_50_ beyond the statistical cutoff but <10-fold mean IC_50_ for the drug were also identified. Sequence data available for nine of these viruses showed the presence of wildtype and mutant sequences at residue 151 of the NA, namely, D151D/G (6 isolates), D151D/N (2 isolates) and D151D/A (1 isolate). Mutations at residue D151 of the NA are associated with reduced susceptibility to zanamivir in A(H3N2) viruses [10], however, these mutations have been shown to be cell culture derived in recent H3N2 viruses [37].

All influenza B viruses tested for oseltamivir (n = 914) were sensitive to the drug with the exception of one outlier B/Texas/38/2008 with an IC_50_ of 12.70 nM; this IC_50_ value was only 4-fold greater than the mean IC_50_ for oseltamivir (3.41 nM) among B viruses tested (Table 3) and 8-fold that of an oseltamivir-sensitive control virus B/Memphis/20/96 (wildtype) (Table 1). This isolate, B/Texas/38/2008, was also an outlier for zanamivir (10.35 nM); its full NA sequence analysis revealed the presence of D197E mutation (corresponding to D198E in N2 numbering). Previously, an influenza B virus with D197E mutation which demonstrated a significant reduction in sensitivity to oseltamivir and zanamivir was detected in a patient with no history of treatment or contact with neuraminidase inhibitors [38].

The influenza B viruses tested for peramivir susceptibility (n = 52) were all sensitive to the drug, while those tested for zanamivir (n = 914) were susceptible to the drug, with the exception of the above-mentioned isolate, B/Texas/38/2008, with the D197E mutation and two other isolates B/New Jersey/02/2009 and B/Okinawa/10/2009 which also exhibited reduced susceptibility to the drug. However, full NA sequence analysis of both B/New Jersey/02/2009 and B/Okinawa/10/2009 did not reveal any changes that may explain the reduced zanamivir susceptibility.

### 2.2. Susceptibility of 2009 pandemic H1N1 viruses to neuraminidase inhibitors

A total of 16 outliers (Figure 1) were identified among the pandemic influenza H1N1 viruses tested for oseltamivir susceptibility (n = 2259), 15 of which were H275Y variants and were characterized as oseltamivir-resistant, while one virus, A/Chile/1579/2009, lacked the H275Y mutation but harbored the mutation I223K (I222K in N2 numbering) in its NA. This isolate, A/Chile/1579/2009, exhibited an IC_50_ for oseltamivir (2.84 nM), which was 12-fold greater than the mean IC_50_ for the drug among H275 wildtype viruses (0.25 nM; Table 4) and 14-fold greater than the IC_50_ for the oseltamivir-sensitive control virus A/California/07/2009 (0.21 nM; Table 1). Of note, full NA sequence analysis of the pandemic isolate, A/Utah/34/2009, with the lowest IC_50_ for oseltamivir among H275Y variants (6.24 nM), revealed the presence of mixed wildtype and mutant sequences at residue H275 (H275H/Y). This isolate was excluded from descriptive statistical analysis of IC_50_s for pandemic H1N1 H275Y variants (Table 4).

All pandemic H1N1 viruses tested for peramivir susceptibility (n = 550) were sensitive to the drug, with the exception of 12 outliers, all of which were H275Y variants.

In contrast to seasonal influenza A(H1N1) viruses, box-and-whisker plot analyses performed on all pandemic influenza H1N1 viruses tested for oseltamivir and peramivir (Figure 1) *versus* that performed only on pandemic H275 wildtype viruses (figure not shown) yielded the same statistical cutoff, since only a few H275Y variants (0.7%) were detected among the pandemic influenza H1N1 viruses as opposed to 93% among seasonal influenza A(H1N1) viruses. Similar to the seasonal influenza A(H1N1) viruses, descriptive statistical analyses of oseltamivir and peramivir IC_50_s for pandemic H1N1 H275Y variants were performed separately from those of H275 wildtype viruses (Table 4), given their distinct genotypes and phenotypes.

All pandemic H1N1 virus isolates tested for zanamivir (n = 2259) were sensitive to the drug, except for a few outliers (n = 11) whose IC_50_s were above the statistical cutoff of 0.69 nM, however, their IC_50_s were <10-fold that of the mean IC_50_ for the drug (0.31 nM), and only one outlier, A/Chile/1579/2009 with an IC_50_ of 0.89 nM showed a change in the NA sequence (I223K). This IC_50_ (0.89 nM) was only 3-fold higher than the mean IC_50_ for zanamivir among this subtype (0.31 nM). There were no apparent differences between the mean IC_50_ of zanamivir for H275Y variants (0.38 nM) and H275 wildtype viruses (0.31 nM) (Table 4).

### 2.3. Challenges of defining neuraminidase inhibitor resistance for surveillance

There is no precise definition of resistance to NAIs in the NI assay because there is currently no established and clinically relevant cutoff IC_50_ value which would separate sensitive viruses from resistant ones. Elevated IC_50_s must be combined with detection of known molecular markers of resistance by conventional sequencing [10,21] or pyrosequencing [29,30] to define NAI resistance.

In this study, seasonal or 2009 pandemic H1N1 viruses initially identified as outliers for oseltamivir based on elevated IC_50_ values, were only characterized as oseltamivir-resistant following pyrosequencing analysis to confirm the presence of the H275Y mutation. Outliers among influenza A(H3N2) viruses were shown to harbor mutations at D151 that were earlier associated with reduced susceptibility to zanamivir [10], however virus variants with mutations at residue D151 in N1 and N2 NAs have been shown to be cell culture selected [26,37], therefore, D151 variants may aptly be reported as NAI-sensitive. It is imperative to confirm the presence of identified molecular markers of resistance in the NA of matching primary clinical specimens by conventional sequencing or pyrosequencing.

Various technical difficulties are associated with determining NAI resistance in influenza viruses. Cell culture-based assays cannot be used for antiviral susceptibility surveillance studies because interpretation of NAI susceptibility in such assays is unreliable [27]. Functional NI assays (chemiluminescent or fluorescent) therefore remain the primary means of monitoring susceptibility of influenza viruses to NAIs. Typically, the fluorescent NI assay generates higher IC_50_ values than the chemiluminescent assay [39] and offers a better discrimination between the IC_50_ values of the mutant and wild type viruses; however, it requires higher virus titers than the chemiluminescent assay. The NI assay’s requirement for cell culture propagated viruses offers challenges in defining NAI resistance as studies have shown that even in the absence of drug pressure, propagation of virus outside of the natural host (*i.e.*, MDCK cells) can lead in some cases, to selection of NA variants with altered susceptibility to NAIs [25,26]. For surveillance purposes, it is easy to define resistance in the NI assay, for extreme outliers. However, additional studies are required to define meaningful cutoff values for NAI susceptibility. The definition of resistance could be harmonized through the sharing of resistant reference virus strains and utilization of similar methods for the assessment of IC_50_ values by various laboratories, especially for viruses with resistance demonstrated in clinical studies.

Setting criteria for NAI resistance based on IC_50_ values requires the identification of outliers, which in this study was done by box-and-whisker plot analyses [8]. Box-and-whisker plots typically display differences between populations without making assumptions about the underlying statistical distribution. In this study, initial box-and-whisker plot analysis on seasonal influenza A/H1N1 viruses was performed without assuming that these viruses consisted of different populations (H275 wildtype and H275Y mutants). After visualizing the distributions of IC_50_s on these plots (Figure 1), it was necessary to perform additional box-and-whisker plot analysis on H275 wildtype viruses to determine whether there were outliers among this population that may have had mutations in the NA other than H275Y. There is no set proportion of obvious outliers allowed for proper box-and-whisker plot analysis as a dataset might have no outliers, one outlier, or several outliers.

Characterization of NAI resistance in the NI assay is further complicated by the differences in ranges of IC_50_ values *between* influenza types/subtypes for oseltamivir, zanamivir and other NAIs such as peramivir, which makes it impossible to compare type/subtype and drug specific data. The IC_50_ values may also be affected by assay conditions and may differ for the same virus between assays. In this study, for example, the mean IC_50_ for oseltamivir among influenza B viruses tested (3.41 nM) was ∼14-fold higher than those of seasonal influenza A(H1N1) H275 wildtype, A(H3N2) and pandemic H1N1 H275 wildtype viruses whose mean IC_50_s for the drug were 0.23, 0.24 and 0.25 nM, respectively (Tables 3 and 4). In addition, the influenza B viruses exhibited mean IC_50_s for zanamivir (3.34 nM) and peramivir (0.56 nM) which were 2- to 10-fold and 3- to 7-fold higher, respectively, than those of the seasonal and pandemic influenza A viruses tested. Lower susceptibility of influenza B viruses to NAIs compared to influenza A viruses was demonstrated in several studies [8,10,21]. Clinical studies [40,41] have shown oseltamivir to be somewhat less effective against influenza B compared to influenza A virus infections in young children, probably due to the reduced sensitivity of type B viruses to oseltamivir.

The IC_50_s for different NAIs also differ *within* each virus type/subtype [20,21,33,42]. It was reported that IC_50_s for influenza B viruses were higher for oseltamivir than for zanamivir [42], but in our study the influenza B viruses exhibited IC_50_s that were comparable for both drugs, 3.41 nM and 3.34 nM, respectively (Table 3), and were in agreement with mean IC_50_s published for B viruses circulating between 2004–2007 using a similar chemiluminescent NI assay [10]. Among the seasonal influenza A(H1N1) viruses, the mean IC_50_ for zanamivir was ∼3-fold higher than that of oseltamivir, similar to previous studies where IC_50_s for zanamivir were slightly higher than for oseltamivir: by 1.2-fold in [10] and 1.5-fold in [21]. The influenza A(H3N2) viruses were less sensitive to zanamivir than oseltamivir, exhibiting a mean IC_50_ for zanamivir 7-fold greater than that of oseltamivir, in agreement with a 4-fold difference [21] and a 6-fold difference published for influenza A(H3N2) viruses circulating between 2004–2007 [10]. The pandemic H1N1 wildtype viruses, on the other hand, exhibited mean IC_50_s for oseltamivir and zanamivir that were quite similar, 0.24 nM and 0.30 nM, respectively. All virus type/subtypes tested for peramivir exhibited IC_50_s for the drug that were 2–8 times lower than those of oseltamivir and zanamivir, with exception of seasonal influenza A(H3N2) viruses whose IC_50_s for oseltamivir (0.24 nM) and peramivir (0.17 nM) were similar.

Wide variations in IC_50_s for oseltamivir were observed among seasonal influenza A(H1N1) and pandemic H1N1 H275Y variants, with the seasonal isolate A/England/412/2008 (14.71 nM) and pandemic isolate A/Utah/34/2009 (6.24 nM) exhibiting the lowest IC_50_s for oseltamivir which were ∼10-fold lower than the mean IC_50_s for the drug among the respective H275Y variants. Both isolates showed mixes of wildtype and mutant nucleotide sequences at residue 275 of the NA (H275H/Y) and were excluded from descriptive statistical analyses of seasonal and pandemic H275Y variants, respectively. The presence of wildtype and mutant virus populations in a sample may affect the accuracy of setting definitive criteria for the detection of NAI resistance [26], although such mixes can evolve into fully resistant dominant variant populations with further passage of virus isolates. By contrast, the seasonal influenza A(H1N1) isolate with the highest IC_50_ for oseltamivir, A/Thailand/1035/2008, had a dominant H275Y mutation in addition to a mix of wildtype and mutant sequences at residue D151 (D151D/G). The IC_50_ for this isolate (1023.68 nM) was ∼10-fold higher than the mean IC_50_ for the drug among seasonal influenza A(H1N1) H275Y variants, stressing further the complexity of defining a precise criterion for NAI resistance based on NI assay IC_50_ values. Hence, when defining criteria for resistance to an NAI such as oseltamivir, only IC_50_ values of viruses lacking the cell selected mutations such as those at residue D151 should be evaluated.

## 3. Experimental Section

### 3.1. Viruses and cells

Seasonal and pandemic influenza viruses collected between October 01, 2008 and September 30, 2009 from various geographic regions of the world were submitted to the WHO Collaborating Center for Surveillance, Epidemiology and Control of Influenza at the Centers for Disease Control and Prevention (CDC) in Atlanta, GA, U.S., and propagated in Madin-Darby canine kidney (MDCK) cells (ATCC, Manassas, VA) that were maintained in Dulbecco’s modified eagle’s medium (DMEM) supplemented with 10% fetal bovine serum (FCS), 50 units/ml penicillin and 50 μg/ml streptomycin (Invitrogen, Carlsbad, CA). Oseltamivir-sensitive and -resistant reference viruses representing each antigenic type and subtype were also propagated in MDCK cells.

### 3.2. NA inhibitors

Oseltamivir carboxylate, the active compound of the ethyl ester prodrug oseltamivir phosphate was supplied by Hoffmann-La Roche (Basel, Switzerland), zanamivir by GlaxoSmithKline (Uxbridge, U.K.) and peramivir by BioCryst Pharmaceuticals (Birmingham, AL).

### 3.3. NA inhibition assays

Susceptibilities of viruses to NAIs were assessed in the chemiluminescent NI assay using the NA-Star™ Kit (Applied Biosystems, Foster City, CA) as previously described [10].

### 3.4. Statistical analysis

Fifty percent inhibitory concentration (IC_50_) values were calculated using JASPR curve fitting software, an in-house program developed at CDC. JASPR is freely available from the CDC and can be obtained through Dr. Larisa Gubareva (lgubareva@cdc.gov). Comparative curve fitting on a subset of viruses was performed by Robosage version 7.31 software (GlaxoSmithKline, Research Triangle Park, NC), an add-in for MS Excel (Microsoft Corp., Redmond, WA). Curve fitting in JASPR was done using the equation: V = Vmax * (1 − ([I] / (Ki + [I]))), where Vmax is the maximum rate of metabolism, [I] is the inhibitor concentration, V is the response being inhibited, and Ki is the IC_50_ for the inhibition curve. Curve fitting in Robosage was done using the equation: y = Vmax*(1−(x/(K+x))), where Vmax is the maximum rate of metabolism, x is the inhibitor concentration, y is the response being inhibited, and K is the IC_50_ for the inhibition curve (*i.e.*, y = 50% Vmax when x = K).

Box-and-whisker plot analyses [8] of log-transformed IC_50_ values were performed for each drug and virus type/subtype using SAS 9.2 software (SAS Institute, NC, U.S.) to determine quartiles and interquartile ranges (IQR) necessary for establishing statistical cutoffs for identification of potentially resistant viruses (outliers). Log-transformation of IC_50_ values was necessary as they are not a normally distributed parameter [8]. The statistical cutoff was set at three times the IQR to the right of the third quartile (*X*_0.75_) [10]. All viruses with IC_50_s outside this cutoff (IC_50_ > *X*_0.75_ + 3 IQR) and with IC_50_ >2-fold but <10-fold the mean IC_50_ for the respective drug for each type/subtype were characterized as mild outliers, while those with IC_50_ > *X*_0.75_ + 3 IQR and ≥10-fold the mean IC_50_ for drug for each respective type/subtype were considered extreme outliers. Outliers were subjected to genetic analysis by pyrosequencing and/or conventional sequencing to detect known or novel markers of NAI resistance. Those harboring previously characterized NA mutations associated with NAI resistance were considered drug-resistant; their descriptive statistics were determined separately.

Descriptive statistics to compute the mean, median and standard deviation (SD), and a one-way analysis of variance were performed on original IC_50_ data (not log transformed), using SAS 9.2 software (SAS Institute, NC, U.S.) for each drug and virus type/subtype, excluding outliers, with statistical significance set at α = 0.05. Virus isolates comprising mixes of both wildtype and variant populations were also excluded from the descriptive statistical analyses.

### 3.5. Pyrosequencing

Viral RNA extraction, reverse transcription PCR (RT-PCR) and pyrosequencing on the PyroMark ID platform (Biotage AB, Uppsala, Sweden) to detect molecular markers of NAI resistance in the NA were performed as previously described [29,30]. Generated sequences were aligned and analyzed using Identifire software (Biotage AB, Uppsala, Sweden).

### 3.6. Sequencing by dideoxy chain termination method

Full sequencing of the NA gene to detect markers of NAI resistance was performed as previously described [10]. Briefly, viral RNAs were extracted from isolates using the MagNA Pure LC or Compact platforms (Roche, Indianapolis, IN). Reverse transcription polymerase chain reaction (RT-PCR) was performed using the SuperScript III One-Step HiFi RT-PCR Kit (Invitrogen). Amplified PCR products were purified using ExoSAP-IT^®^ reagent (USB, Cleveland, OH). Sequence template synthesized with ABI Prism^®^ BigDye™ Terminator Kit (Applied Biosystems, Foster City, CA) and purified using ABI Prism^®^ BigDye™ XTerminator Kit (Applied Biosystems, Foster City, CA). Sequences generated in an ABI PRISM 3730 Genetic Analyzer (Applied Biosystems, Foster City, CA) and analyzed using Lasergene^®^ DNAStar software version 7.0 (DNAStar, Madison, WI, U.S.).

## 4. Conclusions

The IC_50_ values resulting from the NI assay provide valuable information for detection of resistant viruses, but they should not be used to draw a direct correlation with the drug concentrations needed to inhibit virus replication in the infected human host, as clinical data to support such inferences are inadequate. Assessment of NAI susceptibility of virus in the NI assay, supported by NA sequencing in the virus isolate and its matching clinical specimen, provides a reliable and reasonably comprehensive approach to the identification of NAI-resistant isolates for surveillance purposes. However, there is a pressing need to establish a clinically relevant IC_50_ cutoff value which could be used to differentiate statistical outliers from truly resistant viruses. Global surveillance for NAI resistance should be sustained to reflect the impact of seasonal and pandemic of influenza, and also to reflect the increasing use of different NAIs and development of novel NAIs.

## Figures and Tables

**Table 1:** Reference viruses used as controls in the chemiluminescent NI Assay.

**Strain Designation**	**Type/Subtype**	**NA Mutations (Genotype)**	**Oseltamivir Susceptibility**	**Mean IC_50_*^a^* ± SD*^b^*, nM (Fold Difference*^c^*)**		
				**Oseltamivir**	**Zanamivir**	**Peramivir**
A/Washington/10/2008	A (H1N1); Seasonal	WT*^d^*	S*^e^*	0.23±0.07 (1)	0.24±0.08 (1)	0.09±0.02 (1)
A/Florida/21/2008	A (H1N1); Seasonal	H275Y	R*^f^*	94.82±27.04 (386)	0.31±0.08 (1)	11.27±1.36 (134)
A/Washington/01/2007	A (H3N2); Seasonal	WT	S	0.15±0.00 (1)	0.47±0.04 (1)	0.12±0.02 (1)
A/Texas/12/2007	A (H3N2); Seasonal	E119V	R	4.19±0.33 (30)	0.51±0.05 (1)	0.14±0.02 (1)
B/Memphis/20/1996	B; Seasonal	WT	S	1.61±0.21 (1)	1.94±0.21 (1)	0.31±0.06 (1)
B/Memphis/20/1996	B; Seasonal	R152K	R	82.62±2.96 (58)	26.35±13.59 (22)	52.80±3.51 (176)
A/California/07/2009	H1N1; Pandemic	WT	S	0.21±0.03 (1)	0.26±0.04 (1)	0.06±0.02 (1)
A/Texas/48/2009	H1N1; Pandemic	H275Y	R	79.94±0.06 (348)	0.36±0.07 (1)	10.06±0.01 (164)

a Average of 3 independent assays.

b SD, Standard Deviation of IC_50_ values.

c Fold difference in IC_50_ values between mutant and wildtype viruses, by drug and virus type/subtype (IC_50_ of mutant/IC_50_ of wildtype virus).

d WT, Wildtype

e S, Sensitive (Susceptible).

f R, Resistant.

**Table 2:** Comparison of neuraminidase inhibition IC_50_ data generated by JASPR and Robosage software.

**Subtype**	**Isolates analyzed (n)**	**Mean IC_50_*^a^* (±SD)*^b^*, nM**					
		**Oseltamivir**			**Zanamivir**		
		**JASPR**	**Robosage**	***r*^c^**	**JASPR**	**Robosage**	***r***
Seasonal A/H1N1 (H275 wildtype)*^d^*	8	0.22±0.05	0.26±0.07	0.970	0.47±0.34	0.56±0.47	0.999
Seasonal A/H1N1 (H275Y variants)*^e^*	106	113.38±45.63	128.57±55.93	0.986	0.50±0.23	0.58±0.30	0.990
Pandemic H1N1	100	0.21±0.06	0.25±0.06	0.939	0.28±0.10	0.31±0.13	0.988
A/H3N2	125	0.24±0.13	0.27±0.16	0.994	1.30±0.93	1.48±1.08	0.994
B	106	3.56±0.94	3.84±1.05	0.990	3.93±1.32	4.19±1.45	0.995

a Determined in the chemiluminescent neuraminidase inhibition assay.

b SD, standard deviation of IC_50_ values.

c *r*, Pearson correlation coefficient.

d H275 wildtype, oseltamivir-sensitive isolates.

e H275Y variants, oseltamivir-resistant virus isolates.

**Table 3:** Neuraminidase inhibitor susceptibility of seasonal influenza virus isolates (2008–2009).

**NAI**	**Influenza Type/Subtype**	Isolates analyzed (n)*	**IC50 (nM)*^a^***					
			**Range**	**Mean (±SD)***^b^*	**Median**	**IQR***^d^*	**X_0.75_***^e^*	**Statistical Cutoff *^f^***
Oseltamivir	A(H1N1) (H275 wildtype)*^g^*	98	0.10–.0.49	0.23±0.08	0.21	0.10	0.28	0.58
	A(H1N1) (H275Y variants)*^h^*	1430	34.69–1023.68	123.32±65.90	105.13	--	--	--
	A/H3N2	833	0.04–1.38	0.24±0.15	0.20	0.15	0.29	0.74
	B	913	0.59–7.75	3.41±0.99	3.38	1.27	4.01	7.82

Zanamivir	A(H1N1) (H275 wildtype)*^g^*	102	0.13–1.03	0.35±0.15	0.31	0.17	0.54	1.05
	A(H1N1) (H275Y variants)*^h^*	1424	0.07–3.49	0.51±0.27	0.45	--	--	--
	A(H3N2)	812	0.22–4.24	1.23±0.80	0.97	0.91	1.58	4.31
	B	911	0.27–8.77	3.34±1.31	3.27	1.94	4.24	10.06

Peramivir	A(H1N1) (H275 wildtype)	19	0.05–0.17	0.10±0.03	0.10	0.05	0.13	0.28
	A(H1N1) (H275Y variants)	215	2.46–510.57	30.56±66.06	12.95	--	--	--
	A/H3N2	219	0.05–0.94	0.17±0.10	0.14	0.07	0.19	0.40
	B	52	0.24–1.10	0.56±0.18	0.56	0.23	0.68	1.37

a Determined in the chemiluminescent neuraminidase inhibition assay.

* Outliers and mixes (comprising wildtype and variant populations) were excluded from the calculation of mean, SD and median of IC_50_ values.

b SD, Standard Deviation of IC_50_ values.

c Cutoff IC_50_ value for NAI-susceptible viruses, determined by Mean IC_50_+3SD.

d IQR, Interquartile Range.

e X_0.75_, 75^th^ Percentile.

f Statistical cutoff of IC_50_ values for NAI susceptibility, determined by *X*_0.75_ + 3IQR. Outliers with IC_50_ above this cutoff and >10 times the mean IC_50_ for each drug, were characterized as extreme outliers; those with known drug-resistance mutations such as H275Y were classified as resistant and analyzed separately.

g H275 wildtype, oseltamivir-susceptible isolates.

h H275Y variants, oseltamivir-resistant virus isolates.

**Table 4:** Neuraminidase inhibitor susceptibility of 2009 pandemic H1N1 virus isolates (2008–2009 season).

**NAI**	**Pandemic H1N1 virus isolates**	Isolates analyzed (n)*	**IC50 (nM)*^a^***					
			**Range**	**Mean (±SD)*^b^***	**Median**	**IQR***^c^*	**X_0.75_*^d^***	**Statistical Cutoff *^e^***
Oseltamivir	H275 wildtype*^f^*	2243	0.05–1.78	0.25±0.11	0.24	0.08	0.28	0.52
	H275Y variants*^g^*	14	54.21–155.00	87.57±25.53	80.30	--	--	--
Zanamivir	H275 wildtype*^f^*	2233	0.08–1.03	0.31±0.08	0.30	0.11	0.36	0.69
	H275Y variants*^g^*	14	0.27–0.53	0.38±0.08	0.36			
Peramivir	H275 wildtype	538	0.03–0.35	0.08±0.04	0.07	0.04	0.10	0.22
	H275Y variants	11	8.1–12.91	10.40±1.37	10.29	--	--	--

a Determined in the chemiluminescent neuraminidase inhibition assay.

* Outliers and mixes (comprising wildtype and variant populations) were excluded from the calculation of mean, SD and median of IC_50_ values.

b SD, standard deviation of IC_50_ values.

c IQR, interquartile range.

d X_0.75,_ 75^th^ Percentile.

e Statistical cutoff of IC_50_ values for NAI susceptibility, determined by *X*_0.75_ + 3IQR. Outliers with IC_50_ above this cutoff and >10 times the mean IC_50_ for each drug, were characterized as extreme outliers; those with known drug-resistance mutations such as H275Y were classified as resistant and analyzed separately.

f H275 wildtype, oseltamivir-susceptible pandemic virus isolates.

g H275Y variants, oseltamivir-resistant pandemic virus isolates.
