# Correction: Okomo-Adhiambo, M. *et al.* Neuraminidase Inhibitor Susceptibility Testing in Human Influenza Viruses: A Laboratory Surveillance Perspective. *Viruses* 2010, 2, 2269–2289

**DOI:** 10.3390/v3081415

**Published:** 2011-08-12

**Authors:** Margaret Okomo-Adhiambo, Katrina Sleeman, Kristina Ballenger, Ha T. Nguyen, Vasiliy P. Mishin, Tiffany G. Sheu, James Smagala, Yan Li, Alexander I. Klimov, Larisa V. Gubareva

**Affiliations:** 1 Virus Surveillance and Diagnosis Branch, Influenza Division, National Center for Immunization and Respiratory Diseases, Centers for Disease Control and Prevention, 1600 Clifton Rd NE, Mailstop G16, Atlanta, GA 30333, USA; E-Mails: gfv3@cdc.gov (M.O.-A.); hhk6@cdc.gov (K.S.); isx0@cdc.gov (K.B.); hsn7@cdc.gov (H.T.N.); fwc7@cdc.gov (V.P.M.); gbq3@cdc.gov (T.G.S.); gpa1@cdc.gov (J.S.); axk0@cdc.gov (A.I.K.); 2 Atlanta Research and Education Foundation, 1670 Clairmont Rd, 151F, Decatur, GA 30033, USA; 3 Battelle, Century Plaza 1, 2987 Clairmont Rd, Suite 450, Atlanta, GA 30329, USA; 4 Oak Ridge Institute for Science and Education, MC-100-22, P.O. Box 117, Oak Ridge, TN 37831, USA; 5 Influenza and Respiratory Viruses Section, National Microbiology Laboratory, Public Health Agency of Canada, 1015 Arlington St., Suite H4050, Winnipeg, MB, R3E 3R2, Canada; E-Mail: yan.li@phac-aspc.gc.ca

The authors would like to make the following corrections to their published paper:

There was an error in calculation of IC_50_ fold changes for the NAI-resistant viruses reported in Table 1 of the above-mentioned paper. The corrected values are marked in the updated Table 1 below.

We apologize for any inconvenience caused to the readers.

**Table 1.** Reference viruses used as controls in the chemiluminescent NI Assay.

**Strain Designation**	**Type/Subtype**	**NA Mutations (Genotype)**	**Oseltamivir Susceptibility**	**Mean IC_50_*^a^* ± SD*^b^*, nM (Fold Difference *^c^*)**		
				**Oseltamivir**	**Zanamivir**	**Peramivir**
A/Washington/10/2008	A (H1N1); Seasonal	WT*^d^*	S*^e^*	0.23±0.07 (1)	0.24±0.08 (1)	0.09±0.02 (1)
A/Florida/21/2008	A (H1N1); Seasonal	H275Y	R*^f^*	94.82±27.04 ( 386412)	0.31±0.08 (1)	11.27±1.36 ( 134125)
A/Washington/01/2007	A (H3N2); Seasonal	WT	S	0.15±0.00 (1)	0.47±0.04 (1)	0.12±0.02 (1)
A/Texas/12/2007	A (H3N2); Seasonal	E119V	R	4.19±0.33 ( 3028)	0.51±0.05 (1)	0.14±0.02 (1)
B/Memphis/20/1996	B; Seasonal	WT	S	1.61±0.21 (1)	1.94±0.21 (1)	0.31±0.06 (1)
B/Memphis/20/1996	B; Seasonal	R152K	R	82.62±2.96 ( 5851)	26.35±13.59 ( 2214)	52.80±3.51 ( 176170)
A/California/07/2009	H1N1; Pandemic	WT	S	0.21±0.03 (1)	0.26±0.04 (1)	0.06±0.02 (1)
A/Texas/48/2009	H1N1; Pandemic	H275Y	R	79.94±0.06 ( 348381)	0.36±0.07 (1)	10.06±0.01 ( 164168)

a Average of 3 independent assays.

b SD, Standard Deviation of IC_50_ values.

c Fold difference in IC_50_ values between and virus type/subtype (IC_50_of mutant/IC_50_ of wildtype virus).

d WT, Wildtype.

e S, Sensitive (Susceptible).

f R, Resistant.

